# Supplementary material for: Thermophilic Filamentous Fungus C1-Cell-Cloned SARS-CoV-2-Spike-RBD-Subunit-Vaccine Adjuvanted with Aldydrogel®85 Protects K18-hACE2 Mice against Lethal Virus Challenge
Source: Vaccines (Basel). 2022 Dec 11;10(12):2119. doi: 10.3390/vaccines10122119 (PMC9783968; doi:10.3390/vaccines10122119)
Supplement: Supplementary file 1 [file vaccines-10-02119-s001.zip › vaccines-1998728-supplementary.pdf]

Thermophilic Filamentous Fungus C1-Cell-Cloned SARS-CoV-2-Spike-RBD-Subunit-Vaccine Adjuvanted with Aldydrogel®85 Protects K18-hACE2 Mice Against Lethal Virus Challenge.

Supplementary Materials

Table S1. DNA Oligonucleotide primers used in cloning of RBD-C-tag and C1 strain construction.

Assay S1. Quantitative assays for human Angiotensin Converting Enzyme 2 receptor binding reactions between purified recombinant C1-RBD-C-tag and ACE2.

Assay S2. Anti-SARS-CoV-2-RBD-specific IgG monitoring by direct ELISA

Fig S1. The dose responsiveness of C1-RBD-C-tag to recombinant ACE2.

Supplementary tables.

Table S1. DNA Oligonucleotide primers used in cloning of RBD-C-tag and C1 strain construction.

Primer	Usage	Sequence
oMYT1371_Blg8_pr_for	Amplification of fragment from GenScript plasmid, forward	CCTTTCCCTTACTCACACAGACA
oMYT2533_RBD_chi_term_R	Amplification of fragment from GenScript plasmid, reverse	GCTCCCCTCTCTCGACCAGGACTCGGGAGGTCATAGGGCCTTAGGCCTCCGGCTCGCTGC
oMYT0746_bgl8_5int_1	Check for correct integration in genome	CTCGCACCGTAAGGACCAAG
oMYT2823_RBD_start_rev	Check for correct integration in genome	CGTTGAAGACCTCGCCGAA
oMYT1277_trpCtermF	Check for correct integration in genome	GTGAATGCTCCGTAACACCC
oMYT0748_bgl8_3int_1	Check for correct integration in genome	AGCGTGACCCACTCAGGTAA
oMYT0529_bgl_qPCR	Check for clone purity	CACTACGTCAAGTTCGTCGA
oMYT0532_bgl_qPCR	Check for clone purity	GCCGTAGAAGTCGTTGGAG
oMYT0086_bgl_pr_s3	Sequencing the construct	CATCATCCGTCTTCCATCC
oMYT0107_chi1_term_rev	Sequencing the construct	GGTAAACATGTCCAGGCTTC
oMYT0461_03424-1_actin_qPCR	Check for DNA clone purity, reference gene	CGACATGGAGAAGATCTGGC
oMYT0462_03424-1_actin_qPCR	Check for DNA clone purity, reference gene	AGCGTTGAACGTCTCGAAG

.

Supplementary Assays.

Assay S1. Quantitative assays for human Angiotensin Converting Enzyme 2 receptor binding reactions between purified recombinant C1-RBD-C-tag and ACE2.

These binding assays were performed in an ELISA (Enzyme Linked ImmunoSorbent Assay) format. Briefly, 5 µg/ml of purified recombinant ACE2 was used to coat 96-well microtiter plates (Maxisorp, Thermo Fisher Scientific, UK) in 0.1 M carbonate buffer (pH 9.6) at 4°C overnight. Next, the plates were washed three times with wash buffer (0.9% NaCl, 0.05% Tween-20). After blocking non-specific protein binding with blocking buffer, additional buffer was added containing 2% BSA; 0.14M, NaCl; 0.05M, Trizma-base, pH 7.4; 0.05%, Tween-20; 0.05%, NaN_3. Next the ACE2 containing ELISA plates were washed, and then serially diluted RBD-C-tag protein, 20 µg/ml, (and a control reference preparation of RBD manufactured in CHO cells), were added and incubated at 37°C for 1 hour, followed by three washes. RBD-C-tag binding to plate bound ACE2 was detected with purified Rabbit anti-RBD-IgG (IIBR) generated against RBD produced in mammalian cells and alkaline phosphatase-conjugated Goat Anti-Rabbit-IgG (GARIG, Jackson Immuno Research laboratories, Inc., PA, USA, 111-055-003) incubated at 37°C for 1 hr., followed by washing. The ACE2-RBD protein binding reactions were visualized by addition of SIGMAFAST™ p-NitroPhenyl Phosphate substrate (NPP, Sigma-Aldrich, MA USA) at 37°C for 30 minutes and protein absorbances at 405 nm were measured with a SpectraMax iD3 ELISA plate reader (Molecular Devices, CA, USA) fitted with SoftMax v7.0 software.

Assay S2 Anti-SARS-CoV-2-RBD-specific IgG monitoring by direct ELISA.

Blood samples were collected from tail bleedings of vaccinated and naïve mice (BALB/c or K18-hACE2 transgenic mice on the C57BL/6J background) before and after prime and boost vaccination. These blood samples were coagulated at room temperature for 1 hour using Microtainer blood collection SST™ Tubes (BD, NJ, USA) and then centrifuged at 3,000 rpm for 10 min at 4 °C. The upper serum layer was collected and stored at −20°C. Recombinant RBD-C-tag was used to coat 96-well microtiter plates (NUNC-MaxiSorp, Thermo Fisher Scientific, USA) at a final concentration of 2.5 µg/ml in 50 mM carbonate coating buffer, pH 9.6 (Sigma-Aldrich, MA, USA) at 4°C overnight.

The next day, plates were washed three times with (0.9% NaCl, 0.05% Tween-20) and blocked with (2% BSA, 0.14M NaCl, 0.05M Trizma-base pH 7.4, 0.05% Tween-20, 0.05% NaN_3) for 1 hr. at 37°C. Serially diluted mice sera were added and incubated at 37°C for 1 hr., and then the plates were washed three times. Donkey anti-Mouse IgG conjugated to Horseradish Peroxidase (DAMIG-HP, Jackson Immuno Research laboratories, Inc. PA, USA. 715-035-151) was diluted 1:1000 in blocking solution and added to the wells (100 µl/well). After incubation for 1 hr. at 37°C, the plates were washed three times and developed with 3,3′,5,5′-TetraMethylBiphenyldiamine (TMB, Sigma-Aldrich, St. Louis, MA, USA)

for 10 min. The reactions were stopped with 50 μl/well of 1.0 M H_2SO_4 reaction-stop solution. The absorbance was then measured with a microplate reader at 450 nm. To measure the titer of RBD-specific antibodies induced by recombinant protein antigen, peripheral blood serum samples were serially diluted and measured by titration and the Geometric Mean Titer (GMT or Geomean) of RBD-specific IgG in sera was calculated.

Supplementary figures.

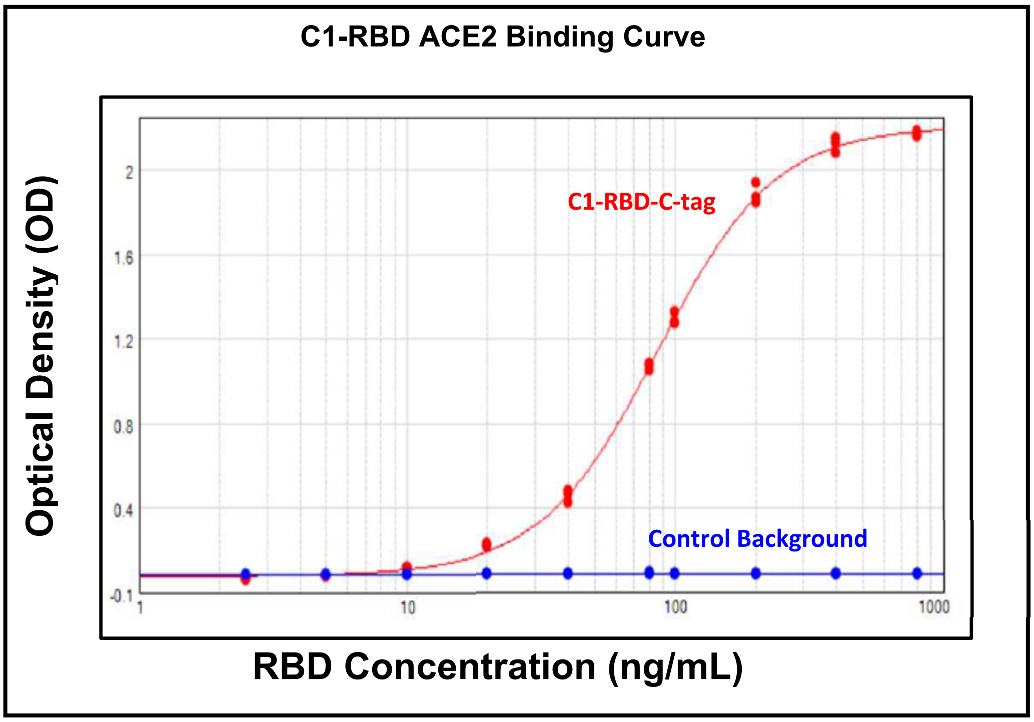

Figure S1. The dose responsiveness of C1-RBD-C-tag to recombinant ACE2.

Detection of C1-RBD-C-tag recombinant ACE2 binding interaction where ACE2 protein was coated on 96-well microtiter plates and C1-RBD concentration was varied, 1000-2.5 ng/ml.
